# The pleiotropic role of p53 in functional/dysfunctional neurons: focus on pathogenesis and diagnosis of Alzheimer’s disease

**DOI:** 10.1186/s13195-020-00732-0

**Published:** 2020-12-03

**Authors:** Giulia Abate, Giovanni B. Frisoni, Jean-Christophe Bourdon, Simona Piccirella, Maurizio Memo, Daniela Uberti

**Affiliations:** 1 Department of Molecular and Translational Medicine, University of Brescia, Viale Europa, 11, 25123 Brescia, BS Italy; 2 Memory Clinic, University Hospitals and University of Geneva, Geneva, Switzerland; 3 School of Medicine, University of Dundee, Dundee, DD1 9SY UK; 4 Diadem srl, Brescia, Italy; 5 Molecular Markers Laboratory, IRCCS Istituto Centro San Giovanni di Dio Fatebenefratelli, Brescia, Italy

**Keywords:** Alzheimer’s disease, p53, Conformational variant of p53

## Abstract

**Background:**

Understanding the earliest pathophysiological changes of Alzheimer’s disease (AD) may aid in the search for timely diagnostic biomarkers and effective disease-modifying therapies. The p53 protein is mostly known for its role in tumor suppression. However, emerging evidence supports that dysregulated p53 activity may contribute to various peripheral and brain alterations during the earliest stages of AD. This review describes the mechanisms through which p53 dysregulation may exacerbate AD pathology and how this could be used as a potential peripheral biomarker for early detection of the disease.

**Main body:**

p53, known as the guardian of the genome, may underlie various compensation or defense mechanisms that prevent neurons from degeneration. These mechanisms include maintenance of redox homeostasis, regulation of inflammation, control of synaptic function, reduction of amyloid β peptides, and inhibition of neuronal cell cycle re-entry. Thereby, dysregulation of p53-dependent compensation mechanisms may contribute to neuronal dysfunction, thus leading to neurodegeneration. Interestingly, a conformational misfolded variant of p53, described in the literature as unfolded p53, which has lost its canonical structure and function, was observed in peripheral cells from mild cognitive impairment (MCI) and AD patients. In AD pathology, this peculiar conformational variant was caused by post-translational modifications rather than mutations as commonly observed in cancer. Although the presence of the conformational variant of p53 in the brain has yet to be formally demonstrated, the plethora of p53-dependent compensation mechanisms underscores that the guardian of the genome may not only be lost in the periphery during AD pathology.

**Conclusion:**

These findings revisit the role of p53 in the early development and exacerbation of AD pathology, both in the brain and periphery. The conformational variant of p53 represents a potential peripheral biomarker that could detect AD at its earliest stages.

## Background

Alzheimer’s disease (AD) spans a clinical continuum from normal cognition to irreversible cognitive impairment, accounting for 60 to 80% of dementias [1, 2]. The disease constitutes a serious health concern in the aging population with a rapidly growing socio-economic burden [3]. The worldwide economic costs associated with dementia, which consist of direct medical, direct social sector, and informal care costs, increased by 35% between 2010 and 2015 and are estimated to rise to USD 2 trillion by 2030 [3]. A growing body of evidence demonstrates that the pathophysiological features of AD have an onset decades before the first symptoms appear [4, 5]. During this preclinical stage, compensation or defense mechanisms, such as the DNA damage response, may initially overcome the alterations in the brain, preventing cognitive decline [6]. Some of these defense mechanisms may be dependent on p53, also known as the guardian of the genome [7]. The role of p53 beyond tumor suppression has been well established in recent years, demonstrating that dysregulated p53 signaling contributes to different pathologies including diabetes, obesity, and aging-associated neurodegenerative disorders [8]. Dependent on the stress stimulus, cell type, and context, p53 regulates the decision between life and death by either inducing apoptosis or by preventing and repairing cellular damage [8, 9]. A non-systematic review of the literature was performed, using the National Library of Medicine’s PubMed database up to October 2020, to collect evidence on the possible role of p53 in the pathogenesis of AD aiming to describe how dysregulated p53 signaling may exacerbate AD pathology, while holding promise as an early predictive peripheral biomarker of the disease.

### AD beyond amyloid and tau pathology

For almost 20 years, the amyloid hypothesis has been regarded as the most important driver of the pathogenesis of AD. According to this hypothesis, amyloid β (Aβ) peptides accumulate during the process of aging forming Aβ fibrils and ultimately senile plaques. This was supported by the fact that in less than 1% of cases, AD is caused by specific mutations in three genes. These genes are coding for amyloid-precursor protein (APP), Presenilin 1 (PS1), and Presenilin 2 (PS2) which are all linked to Aβ metabolism [10]. However, the amyloid hypothesis has come under scrutiny because immunotherapies targeting Aβ failed to improve clinical outcomes in patients with mild-to-moderate AD [11–13]. It is currently unknown, if failure could be attributed to late diagnosis of patients with already severe neuronal loss or if amyloid is not the only target to fight in AD. As demonstrated by positron emission tomography, tau accumulation is pathologically more relevant than the formation of amyloid plaques [10]. In the AD brain, tau becomes hyperphosphorylated leading to the formation of neurofibrillary tangles (NFTs). By destabilizing microtubules, these NFTs cause neuronal cell death and neurodegeneration, correlating with cognitive decline and dementia in AD patients [10]. However, other pathophysiological processes, including accumulation of oxidative stress, induction of a pro-inflammatory environment, and neuronal cell cycle re-entry, may precede the onset of amyloid and tau pathology.

Oxidative stress, which is the result of an imbalance between reactive oxygen species (ROS) and the activity of antioxidant enzymes, was found to be one of the earliest events contributing to synaptic impairment in AD [5]. Aβ and AD risk factors, such as advanced age and apolipoprotein E ε4 (ApoEε4), can all exacerbate oxidative stress [14]. Accordingly, increased markers of oxidative and nitrosative stress have been found in different post-mortem brain regions of patients with AD [15]. Furthermore, oxidative stress can induce tau hyperphosphorylation, suggesting that indeed oxidative stress is an early event in the establishment of neurodegeneration [14, 16].

Besides oxidative stress, the presence of immune-related antigens and cells around amyloid plaques in the brains of patients with AD has been reported since the 1980s [17–19]. Both amyloid and tau pathology may be exacerbated by chronic activation of microglia, astrocytes, and other immune cells. This is further supported by emerging evidence demonstrating that mutations in microglia-specific receptors significantly increase the risk of developing AD [20]. Research is currently aiming to disentangle the exact pathophysiological mechanisms of inflammation in AD to understand how neuroinflammation may predispose patients to develop AD.

Lastly, evidence for neuronal cell cycle re-entry has been known since the mid-1990s when several phospho-epitopes characteristics of dividing cells were discovered in the AD brain but not in age-matched controls [21]. Already during the earliest stages of AD, post-mitotic neurons re-enter the cell cycle, following either apoptosis or DNA synthesis similarly leading to cell death before cell division [22, 23]. The cell cycle hypothesis, therefore, explains the rather slow rate of atrophy during the progression of AD. The chapters below aim to describe how dysregulation of p53 activity may underlie these different pathophysiological mechanisms and compile the evidence that p53 signaling is indeed compromised in patients progressing to AD.

### p53 and its isoforms: regulators of aging and neuroinflammation?

Tight control of p53 activity is paramount for healthy aging. In fact, aberrant regulation of its activity can contribute to a broad spectrum of pathological conditions, spanning from accelerated aging to tumorigenesis due to excessive activation or inactivation, respectively (reviewed in [8, 24, 25]). The pleiotropic role of p53 in health and disease is dependent on the expression of at least 12 protein isoforms in addition to the expression of the full-length p53 protein [26]. Mice studies have shown that the longevity-assurance activity of p53 depends on the levels of an N-terminally truncated p53 isoform (Δ40p53 or p44) [27, 28]. Overexpression of this isoform in mice induced accelerated aging and aggregation of tau in NFTs, which together with hyperactivation of the insulin-like growth factor signaling network caused synaptic deficits and cognitive decline, characteristic of AD [27, 28]. An additional study delineated that the Δ40p53 isoform was generated through alternative splicing in transgenic mice overexpressing the short cytosolic APP intracellular domain (AICD) peptide, which is an important transcriptional regulator of genes involved in AD pathogenesis [29, 30]. Furthermore, studies have shown that this isoform is activated in an age-dependent fashion in the mouse brain coinciding with an increase in phosphorylated tau [28]. As transgenic mouse models only recapitulate parts of the pathogenesis of AD, brain tissues of late-onset sporadic AD (sAD) patients were examined for the presence of the Δ40p53 isoform. Levels of the Δ40p53 isoform were only slightly but not significantly increased in these post-mortem brain tissues [29]. However, results from brain tissues may have been confounded by intrinsic subject variability and severe neurodegeneration, not completely excluding that this p53 isoform may contribute to the formation of NFTs, neurodegeneration, and accelerated aging in patients progressing to AD [29].

More recently, an imbalance between the expression of two different p53 isoforms was found in senescent astrocytes, which were more abundantly present in brain tissues from AD patients compared with controls [26]. These senescent astrocytes expressed higher levels of the senescence-promoting p53β isoform in comparison with the Δ133p53 isoform, which modulates full-length p53 activity. The imbalance between these two isoforms was found to regulate the neurotoxic function of astrocytes by promoting the senescence-associated secretory phenotype which involves secretion of pro-inflammatory cytokines, such as interleukin-6 [26]. Possibly, shifting the balance towards higher activity of the Δ133p53 isoform in astrocytes could induce their neuroprotective function again, inhibiting or delaying the progression of AD [26]. More research will be needed to understand if an imbalance in the normal ratio of p53 and its isoforms may contribute to the development of AD by accelerating aging and exacerbating neuroinflammation.

### Dysregulation of p53-induced adaptive responses in the AD brain

**Fig. 1:**
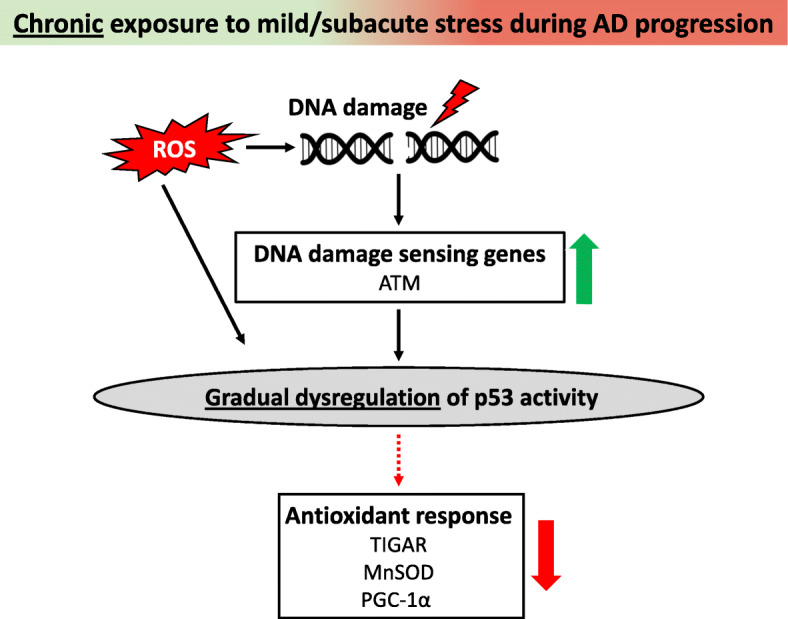
Exacerbation of AD pathology through gradual dysregulation of p53-induced adaptive responses. Upon chronic exposure to mild/subacute stress, p53 becomes activated through ROS or through the induction of DNA damage sensing genes, such as ATM kinase. Following p53 activation, several antioxidant genes (e.g., *TIGAR*, *MnSOD*, and *PGC-1α*) are upregulated to subsequently restore cellular damage. In the AD brain, levels of ATM are strongly upregulated. However, there appears to be a defect in the downstream canonical p53 pathway, as p53-induced adaptive responses are progressively dysregulated during the continuum of the disease, exacerbating AD pathology. AD, Alzheimer’s disease; ATM, ataxia-telangiectasia mutated; MnSOD, manganese superoxide dismutase; PGC-1α, peroxisome proliferator-activated receptor-c coactivator-1a; TIGAR, TP53-induced glycolysis regulatory phosphatase; ROS, reactive oxygen species

Besides polymorphisms and isoforms, the biological function of p53 is mainly regulated by protein-protein interactions and by a complex plethora of post-translational modifications, including phosphorylation, acetylation, and methylation [9]. These post-translational modifications subsequently orchestrate p53-induced adaptive responses, some of which may become gradually dysregulated during the continuum of AD. Upon genotoxic stress, the stability of p53 can be increased by multiple kinases, including ataxia-telangiectasia mutated (ATM) and ATR (ATM Rad3-related) protein kinases, checkpoint kinase 1/2 (Cdk1/Cdk2), and DNA-dependent protein kinase (DNA-PK) [31, 32]. Phosphorylation of p53 by one of the above-mentioned kinases disrupts the p53-mouse double minute 2 (MDM2) interaction, alleviating proteasomal p53 degradation [32]. This cellular defense mechanism becomes activated under conditions of mild or subacute genotoxic stress to promote repair of DNA mutations, which naturally occur during the aging process [31]. Interestingly, microarray analysis revealed that expression of the *ATM* protein kinase was higher in the superior temporal cortex of AD patients with mild (Clinical Dementia Rating [CDR 0.5–1]) and moderate to severe (CDR 2–5) dementia compared with non-demented controls (CDR 0). Accordingly, the gene expression levels of the *ATM* protein kinase correlated with neuritic plaque density and Braak neuropathological stages [33]. The DNA damage response, therefore, appears to be one of the defense mechanisms that is triggered during the progression of AD, resulting in p53 activation (Fig. 1).

DNA damage is often the result of oxidative stress. Under conditions of low and subacute oxidative stress, p53 ensures cell survival by activating several antioxidant genes, such as manganese superoxide dismutase (MnSOD) and *TP53*-induced glycolysis and apoptosis regulator (TIGAR) [31, 34]. However, the p53-induced antioxidant response seems to be impaired in patients with AD. This finding was supported by low MnSOD expression in primary neurons isolated from an AD mouse model carrying mutations in APP and PS1 [35]. As MnSOD is a direct p53 target, this gradual dysregulation of the antioxidant defense during the disease continuum may be caused by impaired p53 activity (Fig. 1).

Additionally, human studies have confirmed that p53-mediated antioxidant responses are altered in post-mortem brain tissues derived from patients with AD. The metabolic pentose phosphate pathway (PPP) is known to be essential for the survival of neurons to cope with elevated production of ROS [36]. As mentioned earlier, p53 can activate TIGAR, which ensures the activity of the PPP, protecting neurons against stress-induced changes [34, 36]. However, the expression of *TIGAR* was inversely correlated with the severity of AD dementia [33]. Although not confirmed in human studies, dysregulation of p53 signaling in the AD brain may be responsible for the downregulation of TIGAR seen in the superior temporal cortex of AD patients with mild (CDR 1) and severe (CDR 5) dementia (Fig. 1).

As mitochondria are the main producers of ROS, maintaining their function and viability is of the utmost importance. p53 can preserve mitochondrial function by regulating mitochondrial biogenesis, removing non-functional mitochondria, maintaining redox homeostasis, and controlling metabolism [36]. Dependent on the cell type and stimulus, p53 can either induce or repress peroxisome proliferator-activated receptor-c coactivator-1a (PGC-1α), which is a known inducer of mitochondrial biogenesis [36]. Interestingly, activation of PGC-1α by p53 was demonstrated in neuroblastoma cells upon depletion of glutathione. Surprisingly, this activation did not increase mitochondrial biogenesis but induced an antioxidant response [37]. This p53-induced antioxidant response may also be neuroprotective in vivo, as glutathione was found to be decreased in the hippocampi of patients with mild cognitive impairment (MCI) (CDR 0.7 and Mini-Mental State Examination [MMSE]: 25.5) and AD (CDR 1.5 and MMSE: 18.4). The study confirmed that this decrease in glutathione was not attributed to tissue atrophy [38]. Furthermore, the reduced glutathione levels significantly correlated with the degree of cognitive impairment. Interestingly, also PGC-1α expression was found to decrease with the severity of dementia when examining the hippocampi of AD patients with different neuritic plaque and Aβ_X-42_ contents. These findings again imply that loss of the p53-induced adaptive response is involved in the exacerbation of oxidative stress early on during the progression of AD [39] (Fig. 1). As a result of increased oxidative stress, pro-inflammatory genes may be induced, shifting the immune balance towards a pro-inflammatory phenotype [40]. Emerging evidence suggests that also p53 itself may modulate the pro-inflammatory environment [41], but the exact implications for AD remain to be deciphered.

Although the above-described evidence suggests that impairment of p53-induced adaptive responses are associated with neurodegeneration observed in AD, the link between the neuroprotective effects of p53 and AD remains controversial. This is mostly due to studies which have demonstrated that p53 is accountable for neurodegeneration due to induction of neuronal apoptosis. In vitro experiments have shown that pro-apoptotic p53 target genes are triggered upon exposure of cells to excessive levels of genotoxic/oxidative stress or micromolar Aβ concentrations [42, 43]. However, neurodegeneration in AD is not the result of a single acute toxic insult and subsequent apoptosis, but rather due to chronic subtoxic oxidative stress insults (reviewed in [44]), resulting in the progressive loss of compensatory mechanisms, such as the p53-induced antioxidant response. Therefore, the role of p53 in AD pathology may need to be revisited, as dysregulation of p53 activity throughout the disease continuum may underlie the oxidative stress hypothesis, which is known to be an early determinant of the pathogenesis of AD [5].

### Revisiting the p53-Aβ feedback loop in the AD brain

**Fig. 2:**
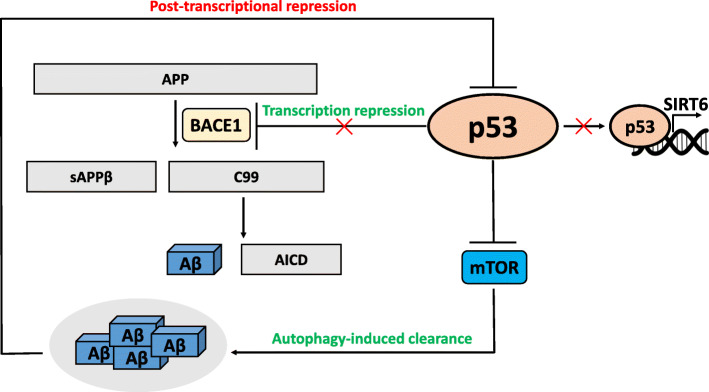
The p53-Aβ feedback loop revisited. The p53 protein inhibits the amyloid cascade by repressing the transcription of BACE1. Additionally, p53 inhibits the mTOR signaling pathway, resulting in Aβ protein clearance through the induction of autophagy. However, Aβ peptides may prevent these neuroprotective p53 effects through posttranscriptional repression of p53, which leads to reduced binding of p53 to the promotor of the anti-aging gene, SIRT6. Aβ, amyloid beta; AICD, amyloid precursor protein intracellular domain; APP, amyloid precursor protein; BACE1, beta-site APP-cleaving enzyme 1; mTOR, mammalian target of rapamycin; sAPPβ, soluble amyloid precursor protein beta; SIRT6, sirtuin-6

Abnormal activity of p53 may also affect other causal paths in the development of AD (Fig. 2). Wild-type p53 is a well-known inhibitor of the mammalian target of rapamycin (mTOR) signaling pathway, which is hyperactivated in the brain and periphery before the AD dementia stage [45–47]. In vitro experiments using primary neuron cultures from mice have shown that hyperactivation of the mTOR signaling axis was in part mediated by the accumulation of Aβ, which activates mTOR complex 1 (mTORC1) at the plasma membrane. Following activation, mTORC1 phosphorylates tau at a serine residue that is required to induce aberrant cell cycle re-entry of post-mitotic neurons [48]. Additionally, a study using an AD transgenic mouse model found that buildup of Aβ increased the activity of the mTOR pathway initiating a negative feedback loop which reduced the clearance of Aβ peptides by inhibiting autophagy [49]. As mentioned earlier, mTOR signaling is also increased in human brain samples of patients affected by amnestic MCI (soluble Aβ_1–42_ concentration: 29.7 fmol/mg and MMSE: 23.3) and AD (soluble Aβ_1–42_ concentration: 35.1 fmol/mg and MMSE: 12.9) in comparison with healthy controls (soluble Aβ_1–42_ concentration: 8.08 fmol/mg and MMSE: 28.7) [50]. Although not formally demonstrated in AD, hyperactivation of the mTOR pathway may be caused by dysregulated p53 signaling. If unfunctional p53 is unable to repress mTOR signaling, this may lead to hyperactivation of the pathway with a concomitant inhibition of autophagy and aberrant neuronal cell cycle, linking impaired p53 signaling to both the amyloid and cell cycle hypothesis. Furthermore, in vitro experiments showed that dysregulated p53 signaling may upregulate beta-site APP-cleaving enzyme 1 (BACE1), which is the rate-limiting enzyme in the generation of Aβ. Interestingly, these in vitro experiments demonstrated that transcription of BACE1 is repressed by p53, protecting against Aβ accumulation [51]. This study, therefore, provided additional evidence that affecting the transcriptional activity of p53 can directly impact the amyloid cascade. Another p53-dependent causal path that may play a role in the development of AD is the downregulation of sirtuin 6 (SIRT6). SIRT6 promotes longevity by controlling DNA repair, genome integrity, energy metabolism, and inflammation [52]. Reduced levels of SIRT6 were observed in human brain samples of AD patients and the underlying mechanism was unveiled in the HT-22 mouse hippocampal neuronal cell line. In this cell line, Aβ_42_ post-transcriptionally reduced p53 protein levels, followed by a decreased binding of p53 to the SIRT6 promotor, downregulating the expression of this anti-aging gene and subsequently compromising longevity [52].

### A conformational change dysregulates p53 signaling in AD

**Fig. 3:**
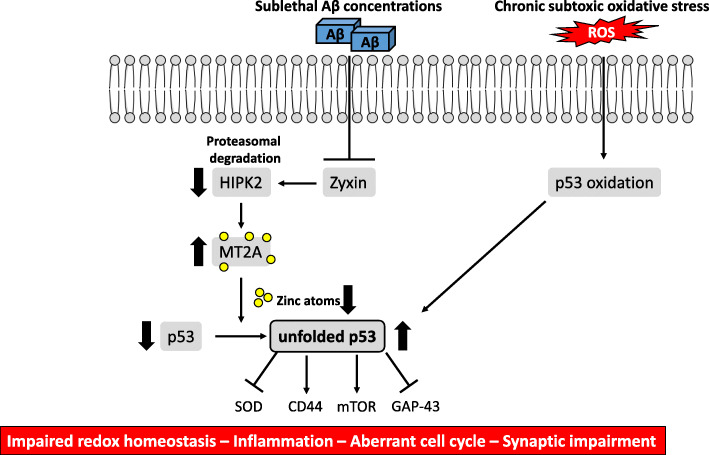
The unfolded p53 protein underlies various early hallmarks of AD pathology. Sublethal Aβ concentrations inhibit the Zyxin/HIPK2 signaling axis, resulting in upregulation of MT2A. Following MT2A upregulation, zinc atoms are chelated which are necessary to maintain wild-type p53 conformation. In addition, chronic exposure to subtoxic nitrosative stress results in nitration of p53 tyrosine residues, misfolding the p53 protein, and dysregulating its activity. An increase in unfolded p53 protein was directly linked to downregulation of SOD and GAP-43, and to upregulation of CD44 and mTOR, potentially leading to impaired redox homeostasis, synaptic impairment, inflammation, and aberrant cell cycle, respectively. Aβ, amyloid beta; GAP-43, growth-associated protein 43; HIPK2, homeodomain interacting protein kinase 2; MT2A, metallothionein 2A; mTOR, mammalian target of rapamycin; ROS, reactive oxygen species; SOD, superoxide dismutase

The above-described evidence is only speculating which downstream p53 effectors could contribute to the progression of AD upon dysregulation of p53 activity. The first evidence to prove that the activity of p53 is indeed altered in AD was found in skin fibroblasts derived from sAD patients [53]. Upon H_2_O_2_ exposure, fibroblasts from sAD patients did not activate canonical p53-dependent cell cycle regulator (*p21* and *GADD45*) and pro-apoptotic (*BAX1*) genes. This impairment resulted in accelerated cell cycle re-entry, leading to diminished H_2_O_2_-induced apoptosis in fibroblasts from sAD patients compared with those from non-AD controls [53]. Although these findings were not confirmed in neurons, they were the first to demonstrate that p53-induced DNA damage repair mechanisms are altered in peripheral cells from AD patients. Lack of p53 activation was caused by a conformational change in its tertiary structure, misfolding the protein and generating a conformational variant of the p53 protein, which is transcriptionally inactive on *p21* and *GADD45* promoters [54]. However, the conformational change was not related to mutations, as commonly is the case in tumor cells [55]. Interestingly, the mutation-independent formation of the conformational variant of the p53 protein was AD-specific, as in vitro models (human embryonic kidney [HEK] and differentiated neuroblastoma SHSY5Y cells) overexpressing the APP_751_ protein and exposure of fibroblasts from non-AD subjects to nanomolar Aβ_1–40_ and Aβ_1–42_ concentrations induced the conformational change in the p53 protein (Fig. 3) [56–58]. Mechanistically, low intracellular amounts of soluble Aβ peptides were shown to deregulate zyxin, which in turn leads to proteasomal degradation of homeodomain interacting protein kinase 2 (HIPK2) [57, 58]. Consequently, downregulation of HIPK2 resulted in the upregulation of metallothionein 2A (MT2A), inducing the conformational variant of p53, which is described in the literature as unfolded p53, by chelating the zinc atom that is responsible for the wild-type p53 conformation [57]. Furthermore, pre-treatment of HEK cells with an Aβ-sequestering antibody or an antioxidant partially prevented the p53 conformational change, implying important roles of both Aβ peptides and the pro-oxidant environment in p53 misfolding [56]. The pro-oxidant environment in immortalized lymphocytes derived from early-onset sAD and familial AD (fAD) patients was shown to induce the p53 conformational change, mainly due to nitration of its tyrosine residues (Fig. 3) [59]. Notably, the increase in unfolded p53 was a marker of impaired redox homeostasis due to its inverse correlation with SOD activity, considering peripheral cells from amnestic MCI (CDR 0.5), severe AD (CDR 2), and healthy subjects (CDR 0) [59, 60]. Concomitant with the increase in unfolded p53, the expression of CD44 was increased in lymphocytes from overt AD patients, which may be of relevance for the immune crosstalk between the periphery and brain in AD pathology [61]. Furthermore, a conformational change in p53 rendered lymphocytes from AD patients less sensitive to rapamycin-mediated inhibition of mTOR signaling, resulting in G (1)/S checkpoint dysfunction [62]. This checkpoint dysfunction could reflect aberrant neuronal cell cycle re-entry and supports the above-described hypothesis that abnormal p53 activity could contribute to dysregulated mTOR signaling in the AD brain.

Although evidence remains scarce, some studies indicate that the conformation of p53 is not only perturbed in peripheral cells but also in the brain regions of AD patients. An experimental study, using neuroblastoma differentiated neuron-like cells overexpressing APP, showed that unfolded p53 downregulated growth-associated protein 43 (GAP-43), which is involved in axonal outgrowth and synaptic plasticity [63, 64]. It is indeed reported in post-mortem brain studies that GAP-43 protein expression is decreased in the frontal cortex of patients with AD [65, 66]. Although these studies did not investigate the p53 pathway, we can speculate that lack of p53 transcriptional activity, due to its conformational changes, is responsible for reduced GAP-43 expression in the AD brain. Additional evidence in post-mortem brain tissues of AD patients showed a huge amount of nitrated and oxidized p53 [67], supporting the findings in immortalized lymphocytes from AD patients. The most compelling evidence for a perturbation of p53 activity in the AD brain was recently demonstrated by Farmer et al. (2020) [68]. This study found that cytosolic p53 aggregates colocalized with tau protein in the frontal cortex of AD patients (Braak stage 6) but not in age-matched controls [68]. It will be interesting to determine in future studies if these p53 aggregates are composed of unfolded p53. Unfolded p53 found in peripheral cells may, therefore, be resembling loss of p53 activity in the AD brain. If this would be the case, unfolded p53 could be a marker of various early AD pathophysiological mechanisms, such as impaired redox homeostasis, neuronal cell cycle re-entry, inflammation, and synaptic deficits (Fig. 3).

### Future perspectives

#### Unfolded p53: a putative peripheral biomarker for early detection of AD

The assessment of biomarkers in peripheral cells and biofluids, such as fibroblasts, lymphocytes, and plasma, offers the advantage to be non-invasive and easily accessible compared to cerebrospinal fluid and imaging biomarkers. A recent review identified that 34% of reports on blood-based biomarkers were related to peripheral changes in emerging mechanisms, such as mitochondrial respiration, inflammatory markers, and oxidative stress response [69]. As described above, the conformational change in p53 may underlie these mechanisms, which may occur early in the disease continuum, supporting the use of unfolded p53 as a putative biomarker for the diagnosis of AD before the onset of cognitive decline. Indeed, initial studies found that the unfolded p53 protein was able to discriminate AD patients from non-AD controls, due to a significantly higher expression of this conformationally altered isoform in peripheral blood mononuclear cells (PBMCs) from overt AD patients (CDR 1.5–2 and MMSE: 17) [70–72]. In addition, the level of unfolded p53 was independent of the APOE ε4 allele carrier status and was also significantly increased in PBMCs derived from preclinical patients with MCI compared with healthy controls. Consequently, elevated levels of the unfolded p53 protein were found to be predictive of the conversion from amnestic MCI to AD dementia [71–73]. Importantly, the unfolded p53 protein was differentially expressed between AD patients and patients affected by either Parkinson’s disease or other types of dementia, confirming that the conformational change in p53 is induced by an AD-specific mechanism [70]. What still limits the general acceptance of p53 involvement in AD is the undeniable reputation it has in cancer. To overcome such view, a new antibody 2D3A8 able to identify a p53 misfolded conformational variant that was found to be highly expressed in AD has recently been developed [74]. This novel 2D3A8 antibody recognizes a linear epitope between the DNA binding domain and the conjunction region with the tetramerization domain. This epitope is less accessible when the protein is assembled in its native form but most likely becomes exposed due to redox post-translation modifications, as suggested by the pro-oxidant environment in AD [74]. In addition, preliminary studies found that the level of unfolded p53 protein detected by the 2D3A8 antibody in PBMCs correlated with age and cognitive impairment in AD [74]. Future longitudinal studies should aim to address whether this peripheral biomarker correlates with the deposition of amyloid and tau in the AD brain and confirm if unfolded p53 is able to diagnose AD at its early stages, predicting if patients with MCI are predisposed to develop AD.

#### Unfolded p53: a potential target for therapeutic monitoring in AD

Considering the oxidative stress hypothesis as one of the earliest mechanisms underlying AD, antioxidant therapies have been studied for years. However, randomized clinical trials examining the clinical benefits of antioxidants, such as vitamin E supplementation, remain inconclusive. Despite the fact that vitamin E is significantly lower in plasma and cerebrospinal fluid of patients with AD compared to those without, vitamin E supplementation did not consistently halt cognitive decline in human trials. One of the caveats of antioxidant supplementation is the identification of appropriate oxidative stress markers for evaluation of its response [75]. As the unfolded p53 conformation is induced by the pro-oxidant environment [59], it has the potential to be one of these oxidative stress markers that can be used for therapeutic monitoring in studies evaluating the impact of antioxidant therapies on the disease progression of AD. In fact, in vitro studies have shown that the formation of the unfolded p53 conformation is a dynamic process that is reversible by restoring the pro-oxidant environment [56]. Therefore, it would be interesting to examine how disease-modifying drugs may influence the conformation of p53 in the periphery and ultimately the brain of AD patients. Additionally, the unfolded p53 conformation was reverted into its wild-type state upon supplementation of zinc to AD fibroblasts [57]. Although rescue of p53 conformational changes upon pharmacological and/or immunological treatment has not been demonstrated in AD, zinc supplementation can reactivate misfolded p53 in cancer, making it a potential cancer therapeutic [76]. Similarly, future studies may investigate whether restoration of p53 conformation could overcome the dysregulated p53 signaling in the brain potentially preventing or delaying AD progression.

## Conclusions

The balance between the harmful and guarding effects of p53 is disturbed in various pathologies, including AD where conformational changes misfold the p53 protein altering its activity. Although the ramifications of p53 activities in the brain remain to be deciphered, emerging evidence suggests that dysregulation of its activity may contribute to various aspects of the pathogenesis of AD. It is becoming well-established that AD manifests beyond the brain, with alterations in the periphery that could aid in the search for biomarkers of the disease. The conformational variant of the p53 protein, known as unfolded p53, could be one of these peripheral biomarkers, which level was significantly higher in peripheral cells derived from patients with AD compared with non-demented controls, Parkinson’s disease, and other types of dementia. In addition, misfolding of the p53 protein was found to expose an AD-specific epitope early on in the disease continuum, holding potential to predict the conversion from MCI to AD. In summary, a conformational change in the p53 protein may contribute to AD exacerbation but holds promise as a potential peripheral biomarker for early detection of AD.

## Data Availability

Not applicable.

## Abbreviations

Aβ: Amyloid beta
AD: Alzheimer’s disease
AICD: Amyloid precursor protein intracellular domain
APOE ε4: Apolipoprotein E ε4 allele
APP: Amyloid precursor protein
ATM: Ataxia-telangiectasia mutated
ATR: ATM Rad3-related
BACE1: Beta-site APP-cleaving enzyme 1
Cdk1/2: Checkpoint kinase 1/2
CDR: Clinical Dementia Rating
DNA-PK: DNA protein kinase
GADD45: Growth arrest and DNA damage-inducible 45
fAD: Familial Alzheimer’s disease
GAP-43: Growth-associated protein 43
HIPK2: Homeodomain interacting protein kinase 2
MCI: Mild cognitive impairment
MDM2: Mouse double minute 2
MMSE: Mini-Mental State Examination
MnSOD: Manganese superoxide dismutase
MT2A: Metallothionein 2A
mTOR: Mammalian target of rapamycin
NFTs: Neurofibrillary tangles
PBMC: Peripheral blood mononuclear cells
PGC-1α: Peroxisome proliferator-activated receptor-c coactivator-1a
PPP: Pentose phosphate pathway
PS: Presenilin
ROS: Reactive oxygen species
sAPPβ: Soluble amyloid precursor protein beta
sAD: Sporadic Alzheimer’s disease
SIRT6: Sirtuin-6
SOD: Superoxide dismutase
TIGAR: TP53-induced glycolysis regulatory phosphatase
